# Self-Recognition of One's Own Fall Recruits the Genuine Bodily Crisis-Related Brain Activity

**DOI:** 10.1371/journal.pone.0115303

**Published:** 2014-12-19

**Authors:** Tomoaki Atomi, Madoka Noriuchi, Kentaro Oba, Yoriko Atomi, Yoshiaki Kikuchi

**Affiliations:** 1 Department of Frontier Health Science, Division of Human Health Sciences, Graduate School of Tokyo Metropolitan University, Tokyo, Japan; 2 Department of Physical Therapy, Faculty of Medical Sciences, Teikyo University of Science, Uenohara, Japan; 3 Division of Medical Neuroimage Analysis, Department of Community Medical Supports, Tohoku Medical Megabank Organization, Tohoku University, Sendai, Japan; 4 Department of Material Health Science, Faculty and Graduate School of Engineering, Tokyo University of Agriculture and Technology, Tokyo, Japan; National University of Singapore, Singapore

## Abstract

While bipedalism is a fundamental evolutionary adaptation thought to be essential for the development of the human brain, the erect body is always an inch or two away from falling. Although the neural mechanism for automatically detecting one's own body instability is an important consideration, there have thus far been few functional neuroimaging studies because of the restrictions placed on participants' movements. Here, we used functional magnetic resonance imaging to investigate the neural substrate underlying whole body instability, based on the self-recognition paradigm that uses video stimuli consisting of one's own and others' whole bodies depicted in stable and unstable states. Analyses revealed significant activity in the regions which would be activated during genuine unstable bodily states: The right parieto-insular vestibular cortex, inferior frontal junction, posterior insula and parabrachial nucleus. We argue that these right-lateralized cortical and brainstem regions mediate vestibular information processing for detection of vestibular anomalies, defensive motor responding in which the necessary motor responses are automatically prepared/simulated to protect one's own body, and sympathetic activity as a form of alarm response during whole body instability.

## Introduction

Bipedalism is the fundamental evolutionary adaptation that sets hominids – and therefore humans – apart from other primates. The human body is arranged vertically, such that the head, trunk, legs, and feet, as well as their links in the neck, spine, pelvis, knees, and ankles, dynamically balance together to form an upright “antigravity pole”. Because these segments and their points of articulation are not fixed, and given that the downward force of gravity never stops, the erect body always exists an inch or two away from falling. Some of the most important brain systems are dedicated to the maintenance of balance against the pull of gravity and to providing an online representation of where the body is located, via the integration of many different exteroceptive/interoceptive inputs (visual, auditory, vestibular, somatosensory, motor, visceral, and so on) [1], [2], [3], [4], [5]. The neural system for rapid detection of potential falls and corresponding automatic reactions to prevent such falls is highly important for human beings, and such a system constitutes one of the most important functions of the body schema, the innate bodily representation system that provides a repertoire of motor functions for promoting survival at the most basic level. The body schema is a plastic and dynamic representation of the spatial and biomechanical properties of the body that is derived from multiple sensory inputs that interact with motor systems [6], [7] and comprises the automatic motor and postural schemata upon which non-conscious movements are based, although these schemata can enter into and support intentional activity [8], [9], [10]. Although investigations of the neural mechanism that prevents us from falling would seem to be important for improving our understanding of basic evolutionary brain structures that support survival, brain scanning technologies such as functional magnetic resonance imaging (fMRI) place major restrictions on participants' movements and thus do not permit study of in-vivo brain activity during falls, near-falls, or other instances of body instability. Here, we explored the possibility of measuring such brain activity by having participants view images of their own bodies in unstable states.

When we see another person's bodily movements associated with emotion, we immediately know what specific movement is associated with a particular emotion, as Darwin argued that emotions are adaptive in the sense that they prompt an action that is beneficial to the organism given its environmental circumstances [11]. A shared representation mechanism based on the body-schema is proposed as the basis for both action [3], [12], [13], [14] and emotion recognition [13], [15], [16], suggesting an intrinsic link between the two. Moreover, self-stimuli show a perceptual advantage in visual recognition, when recognition of one's own body is compared to that of someone else's [17], [18], [19], [20], [21]. These self-stimuli appear to recruit specific underlying neural substrates [22], [23], [24], [25]. Such findings indicate that one's own body sustains a distinct internal representation and that the perception-action matching system is optimally tuned for the observation of one's own actions. We would therefore expect that the internal representation of one's own movements and associated interoceptive representations, which are essential for survival, would be more activated while viewing images of one's own body (from the third person perspective) in an unstable state as compared to viewing the bodies of others. We further reasoned that the brain activity observed while viewing such images would closely approximate that which occurs in response to in-vivo body instability (e.g., slipping suddenly and almost falling down).

We conducted an fMRI experiment using video stimuli consisting of three kinds of whole-body movement: Statically stable (SS), dynamically stable (DS), and dynamically unstable (DU). All three categories of stimulus depicted both the self and unfamiliar individuals. For the present purpose of using fMRI to identify brain activity associated with awareness of body instability, we defined “body instability”, or the unstable components of whole body movements, as the differential visual information based on the subtraction of DS (predictable and stable movements) from DU (unpredictable and unstable movements). Then, our goal was to clarify the nature of any survival-related self-specific activity pertaining to body instability, by directly comparing brain activation associated with the processing of one's own body instability with such activity while viewing others. We hypothesized that such self-specific activity would consist of activity associated with vestibular/interoceptive and defensive processes.

## Materials and Methods

### Participants

Thirteen healthy male participants (mean age  =  24.7±4.3 years) took part in the experiment. All participants were right handed according to the Chapman test (13.3±0.6) and had no neuromuscular diseases. All participants gave their informed consent to participate in the present study.

### Ethics statement

This study was approved by the Research Ethics Committee of Tokyo Metropolitan University, and all participants provided written informed consent to participate in this study. The individual in this manuscript has given written informed consent (as outlined in PLOS consent form) to publish these case details.

### Stimuli, task protocol and procedure

**Figure 1:**
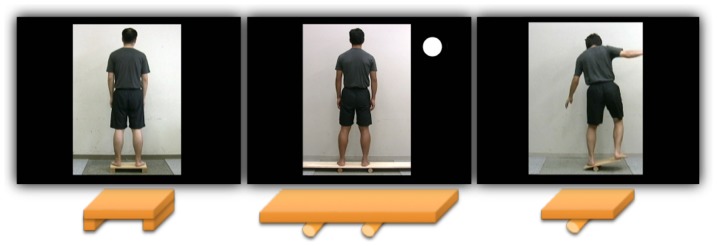
Three kinds of wooden balance boards used in the present experiment. The participant was instructed to stand and maintain his balance on three kinds of wooden balance boards: Two quadrangular pillars (statically stable) (left), two round pillars (dynamically stable) (middle), and the one round pillar (dynamically unstable) (right). A white circle was marked on the right above the self clip.

The stimuli were video clips of the participants' own bodies as well as four other unfamiliar individuals, across the three different conditions described earlier: Statically stable (SS), dynamically stable (DS), and dynamically unstable (DU). Each participant was instructed to stand and maintain their balance on three kinds of wooden balance boards with two quadrangular pillars (6 cm in height, SS), two round pillars (6 cm in diameter, DS), and one round pillar (6 cm in diameter, DU) (Fig. 1). We made recordings of each participant using a digital video camera (HDV10, Cannon) about a month before the fMRI experiment took place. These clips were used as the stimuli during the fMRI experiment. We video-recorded each participant's back for about three minutes in each condition. During all the conditions the participant was instructed to stand at the center of board with his feet shoulder-width apart and upper arms in a natural position, to fixate on a point placed at eye level, and to maintain their balance while minimizing head and trunk movements as much as possible. In the DS condition, the board was moved horizontally at cycles of about 0.27±0.03 Hz and with a range of about 10 cm. In the DU condition, the participant was instructed to keep the board horizontal as much as possible, after having viewed a video demonstration of successful task performance. We recorded the video clips in the same room and place, and participants were wearing the same T-shirt across all conditions, to render the video stimuli as visually comparable across the conditions as possible. Four different clips were extracted from the videos for use in each condition. The clip was edited such that the whole body and board could be seen, with these images surrounded by a black background (Fig. 1). The video clips for the DU condition were edited so that scenes where the participant fell down and made hand or foot contact with the floor were not included. Clips depicting the self were identified as such using a white mark positioned to the right above the image (Fig. 1). A block-design paradigm was applied, with 24 different stimuli (four self and four other clips in each of the 3 conditions) for 32 sec each, with 8 sec rest periods during which a white fixation cross was shown at the center of a black background. The stimuli were projected onto an acrylic screen from the back, with the participant viewing them through a mirror. The distance from the participant's eye to the screen was 228 cm and the size of presented images was 30.5 cm×42.5 cm. The participants were instructed to concentrate on viewing the stimuli without thinking about any other specific things.

### fMRI data analysis

Magnetic resonance imaging data were acquired using a 1.5-T MRI (Signa Horizon LX, GE Medical Systems, Milwaukee, Wisconsin). Changes in blood oxygenation level-dependent T2-weighted magnetic resonance (MR) signals were measured using a gradient echo-planar imaging (EPI) sequence (repetition time [TR]  = 4000 msec, echo time [TE]  = 90.5 msec, field of view [FOV]  =  24×24 cm^2^, flip angle  = 80 degree, 128×128 matrix, 20 slices per volume, slice thickness  =  7.0 mm). The scanning session lasted 968 sec for each participant. A total of 242 EPI volume images were acquired during each scan session, and the first two volumes of each run were discarded because of magnetization instability. We obtained a total of 240 EPI volumes per participant for analysis. Image processing was carried out using Statistical Parametric Mapping software (SPM2, Wellcome Department of Imaging Neuroscience, London, UK; http://www.fil.ion.ucl.ac.uk/spm/software/spm2). The EPI images were realigned and normalized based on the MNI (Montreal Neurological Institute) stereotactic space, and resampled to 2×2×2 mm^3^. The normalized images were smoothed using an 8-mm full-width half-maximum Gaussian kernel. The data were temporally convolved with the hemodynamic response function (HRF) and high-pass filtered with a cutoff period of 128 sec. Each combination of performer (self, others) × condition (DU, DS, SS) was modeled using a separate regressor for each participant. Random effects analysis was then performed at *p*<.001 uncorrected and a cluster size of ≥10. This double threshold corresponds to a 5% multiple comparisons adjusted probability of falsely identifying one or more activated voxel clusters on the basis of Monte Carlo simulations (Alphasim/AFNI (http://afni.nimh.nih.gov/afni/doc/manual/AlphaSim)). We then contrasted brain activity during the DU and DS conditions, separately for self and other (Self DU vs. Self DS, Others DU vs. Others DS) to investigate the neural basis of visual information processing of body instability, and these contrasts were also directly compared (Self (DU vs. DS) vs. Others (DU vs. DS)) to investigate self-specific neural processes related to body instability. In addition, we here defined the “self-specific neural activity” related to body instability as follows: the activity in which it is significantly activated for the contrast of (Self (DU vs. DS) vs. Others (DU vs. DS)) and the averaged eigenvariates in the spherical ROI (radius, 5 mm) centered at each cluster showing significant activity in the above contrast is positive (activation) in the contrast of Self (DU vs. DS). Although there may be some positive effects on the contrast of (Self (DU vs. DS) vs. Others (DU vs. DS)) by deactivation (negative value) in the contrast of Others (DU vs. DS), such deactivation in the Others' condition is also an important aspect of the self-specific neural activity related to body instability in the present study. In fact, it is well known that non-self-referential stimuli induce prominent deactivation in some brain regions such as the cortical midline structures and demonstrate increases in activity during the processing of self-referential stimuli [26]. So we checked whether each of the ROIs in the contrast of Self (DU vs. DS) was positive or not. Furthermore, among the brain regions significantly activated in the contrast, we investigated the possible regions corresponding to the genuine bodily instability based on the previous related studies. In addition, we used forward stepwise selection to assess the relationship between the self-specific neural activity and the differential subjective ratings (see below). We conducted multiple regression analyses with the eigenvariate values in the spherical region of interest (ROI; radius, 5 mm) as the dependent variable, the center of which was the peak voxel in each cluster showing significant activity in the Self (DU vs. DS) vs. Others (DU vs. DS) contrast, and eight of the subjective ratings in which “body stability” and “static state” were excluded because of their high correlation with “body instability” and “dynamic state” respectively, as independent variables. Moreover, we checked the residuals by performing the Kolmogorov-Smirnov test of normality (*p*<0.05), and calculated the Durbin-Watson static for the null hypothesis of no autocorrelation.

### Statistical analysis of subjective ratings

After the fMRI scans, the participants were asked to rate their emotional state while viewing the sample video clips. The sample video clips consisted of 15 clips (the participant's own and four other individuals in each condition), which were selected from the stimuli that had been presented to the participants during the fMRI session. Four items measuring aspects of motion pattern and six items assessing various aspects of emotion were administered as follows: “How much did you feel the body was unstable (body instability), stable (body stability), dynamic (dynamic state) and static (static state)?”, and “How much did you feel anxious (anxiety), relieved (relief), in danger (danger), safe (safety), impatient (impatience) and calm (calmness)?”. We used five-point Likert scales for data collection (“not at all, 0”, and “completely agree, 4”). Statistical analysis was carried out using SPSS version 21.0 software (SPSS, Inc., Chicago, IL). A two-way repeated measures ANOVA (2 performers ×3 conditions) were performed for each of the subjective ratings at *p*<0.01. If the sphericity assumption was violated (significant results in Mauchly's test of sphericity), degrees of freedom were corrected using Greenhouse-Geisser estimates of sphericity. Post-hoc test with Bonferroni correction for multiple comparisons were applied at *p*<0.01. In addition, for each of the subjective ratings, the differential score between Self DU vs. Self DS was compared with that for Others DU vs. Others DS using paired t-tests (*p*<0.01).

## Results

### Subjective ratings

**Figure 2:**
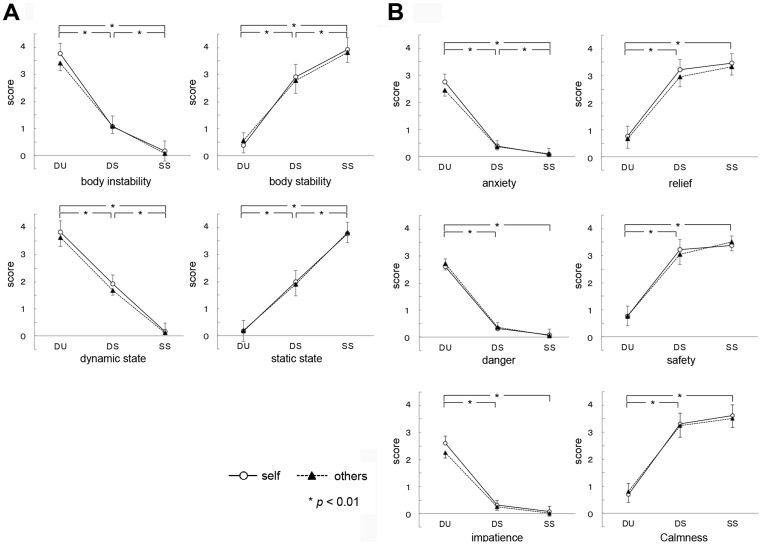
Subjective ratings of motion pattern (A) and emotion (B). There were significant main effects of conditions (DU, DS, and SS), and no significant main effects of performers. DU: dynamically unstable, DS: dynamically stable, SS: statically stable.

In the aspects of motion pattern (Table 1, Fig. 2A), there were no significant interactions between performer and condition, in the body instability (*F* (1.28, 15.31)  = 1.80, *p* = 0.20, Greenhouse-Geisser *ε* = 0.64), body stability (*F* (2, 24)  = 1.08, *p* = 0.36), dynamic state (*F* (2, 24)  = 1.25, *p* = 0.31), and static state (*F* (1.35, 16.18)  = 0.39, *p* = 0.61, Greenhouse-Geisser *ε* = 0.67). There were significant main effects of condition, in all the motion aspects (body instability, *F* (2, 24)  = 223.96, *p* = 0.00; body stability, *F* (2, 24)  = 276.62, *p* = 0.00; dynamic state, *F* (2, 24)  = 166.23, *p* = 0.00; static state, *F* (2, 24)  = 171.13, *p* = 0.00). There were no significant main effect of performer in the body instability (*F* (1, 12)  = 2.02, *p* = 0.18), body stability (*F* (1, 12)  = 0.035, *p* = 0.86), dynamic state (*F* (1, 12)  = 7.26, *p* = 0.020) and static state (*F* (1, 12)  = 0.0070, *p* = 0.93). Multiple comparisons for subjective ratings of motion pattern indicated that participants felt more unstable and dynamic, as well as less stable and static, in the DU condition compared to each of the DS and SS conditions, and in the DS as compared to the SS condition. Moreover, paired-t tests showed that there were no significant differences between the self and others for the DU vs. DS contrast, in body instability (*t* = 1.59, *p* = 0.14), body stability (*t* = −1.29, *p* = 0.22), dynamic state (*t* = −0.24, *p* = 0.82) and static state (*t* = −0.67, *p* = 0.52).

**Table 1:** Results of subjective ratings.

	Self	Others
	DU	DS	SS	DU	DS	SS
body instability	3.77±0.39	1.08±0.28	0.15±0.28	3.42±0.28	1.10±0.11	0.08±0.06
body stability	0.38±0.45	2.92±0.45	3.92±0.44	0.56±0.45	2.79±0.48	3.83±0.38
dynamic state	3.85±0.41	1.92±0.32	0.15±0.31	3.63±0.32	1.67±0.17	0.12±0.07
static state	0.15±0.42	2.00±0.41	3.77±0.41	0.19±0.41	1.90±0.43	3.81±0.37
anxiety	2.77±0.28	0.38±0.21	0.08±0.21	2.44±0.21	0.37±0.13	0.10±0.12
relief	0.77±0.37	3.23±0.36	3.46±0.35	0.67±0.36	2.96±0.37	3.33±0.30
danger	2.62±0.29	0.31±0.23	0.08±0.23	2.71±0.23	0.37±0.10	0.06±0.10
safety	0.77±0.37	3.23±0.37	3.38±0.36	0.77±0.37	3.06±0.39	3.50±0.32
impatience	2.62±0.27	0.31±0.20	0.08±0.20	2.25±0.20	0.25±0.13	0.02±0.11
calmness	0.69±0.41	3.31±0.41	3.62±0.39	0.81±0.41	3.25±0.44	3.50±0.32
						Mean ± S.E.

Mean scores and standard errors (S.E.) for subjective ratings reflecting motion pattern (body instability, body stability, dynamic state, static state) and emotion (anxiety, relief, danger, safety, impatience, calmness).

DU: dynamically unstable, DS: dynamically stable, SS: statically stable.

In the aspects of emotion (Table 1, Fig. 2B), there were no significant interactions between performer and condition, in the anxiety (*F*(1.32, 15.78)  = 1.34, *p* = 0.28, Greenhouse-Geisser ε = 0.66), relief (*F* (2, 24)  = 0.36, *p* = 0.70), danger (*F* (1.27, 15.29)  = 0.19, *p* = 0.73, Greenhouse-Geisser, *ε* = 0.64), safety (*F* (2, 24)  = 1.72, *p* = 0.20), calmeness (*F* (2, 24)  = 0.74, *p* = 0.49), and impatience (*F* (1.33, 15.94)  = 0.81, *p* = 0.41, Greenhouse-Geisser *ε* = 0.66). There were significant main effects of condition, in all the aspects of emotion (anxiety, *F* (2, 24)  = 82.99, *p* = 0.00; relief, *F* (1.28, 15.40)  = 65.91, *p* = 0.00, Greenhouse-Geisser *ε* = 0.64; danger, *F* (1.22, 14.67)  = 108.50, *p* = 0.00, Greenhouse-Geisser *ε* = 0.61; safety, *F* (1.21, 14.48)  = 54.79, *p* = 0.00, Greenhouse-Geisser *ε* = 0.60; calmness, *F* (1.32, 15.85)  = 116.35, *p* = 0.00, Greenhouse-Geisser, *ε* = 0.66; impatience, *F* (1.14, 13.62)  = 96.58, *p* = 0.00, Greenhouse-Geisser *ε* = 0.57). There were no significant main effect of performer in the relief (*F* (1, 12)  = 7.61, *p* = 0.017), calmness (*F* (1, 12)  = 0.026, *p* = 0.017), anxiety (*F* (1, 12)  = 0.85, *p* = 0.38), danger (*F* (1, 12)  = 0.34, *p* = 0.57), safety (*F* (1, 12)  = 0.038, *p* = 0.85) and impatience (*F* (1, 12)  = 4.34, *p* = 0.059). Multiple comparisons for subjective ratings of emotion indicated that participants felt more anxious, in danger, and impatient, as well as less relieved, safe and calm, in the DU condition compared with each of the DS and SS conditions, and that they felt more anxious in the DS compared with the SS conditions. Moreover, paired-t tests showed that there were no significant differences between the self and others for the DU vs. DS contrast, in anxiety (*t* = 1.03, *p* = 0.33), relief (*t* = −0.67, *p* = 0.51), danger (*t* = −0.16, *p* = 0.88), safety (*t* = −1.00, *p* = 0.34), impatience (*t* = 0.91, *p* = 0.38) and calmness (*t* = −0.84, *p* = 0.42).

### Neural activity in the DU vs. DS contrast for self and others

**Figure 3:**
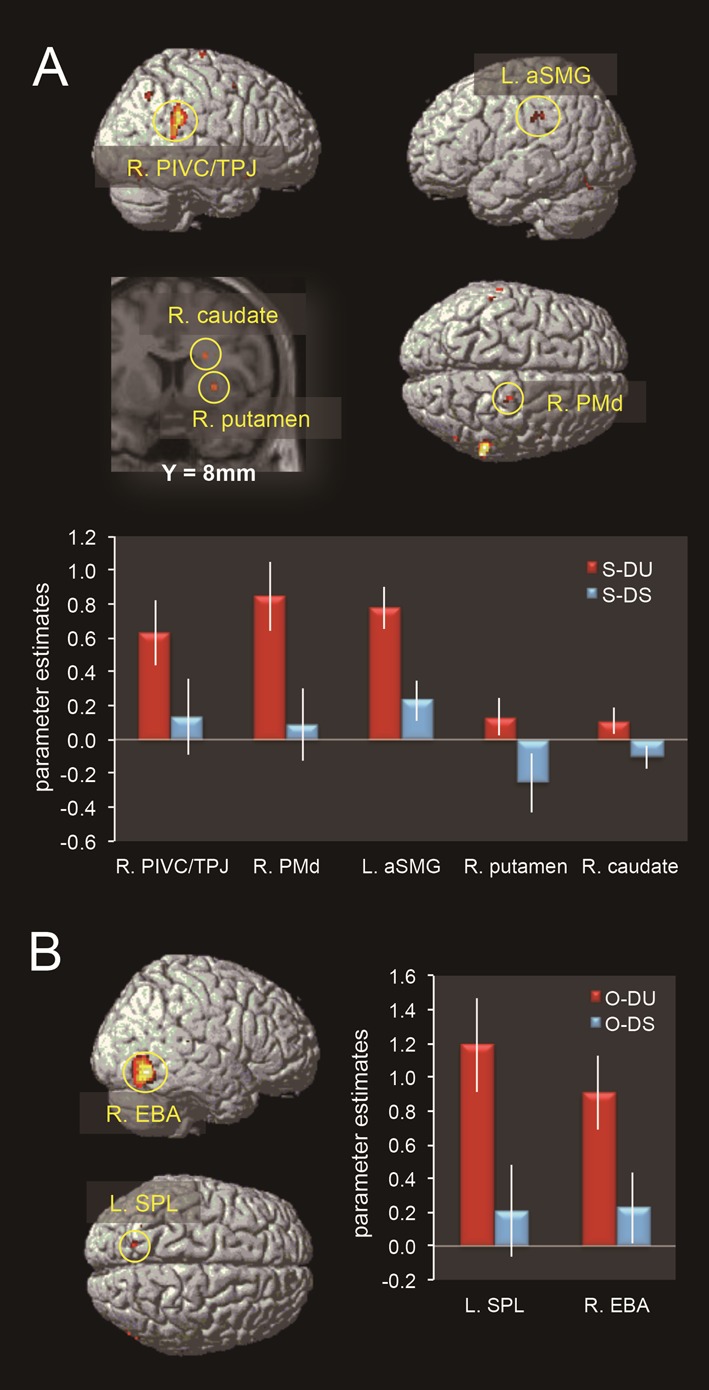
Brain activity in the DU vs. DS contrast for self and others. **A:** Brain regions significantly activated for the Self DU vs. Self DS contrast, and the eigenvariate values (parameter estimates, mean ± standard error) in the spherical region of interest (ROI; radius, 5 mm) whose center was the peak voxel at each cluster showing significant activity in the above contrast, in each of the Self DU (S-DU) and Self DS (S-DS) comparisons. **B:** Brain regions significantly activated for the Others DU vs. Others DS contrast, and the eigenvariate values in the spherical ROI (radius, 5 mm) whose center was the peak voxel at each cluster showing significant activity in the above contrast, in each of the Others DU (O-DU) and Others DS (O-DS) comparisons. R: right, L: left, PMd: dorsal premotor area, PIVC: parieto-insular vestibular cortex, TPJ: temporo-parietal junction, IPL: inferior parietal lobe, aSMG: anterior supramarginal gyrus, EBA: extrastriate body area, SPL: superior parietal lobe.

As shown in Table 2 and Fig. 3A, the Self DU vs. Self DS contrast revealed activation in the right dorsal premotor cortex (PMd), parieto-insular vestibular cortex (PIVC)/temporo-parietal junction (TPJ), inferior parietal lobe (IPL), fusiform gyrus, putamen and caudate nucleus, left anterior supramarginal gyrus (aSMG), and the fusiform gyrus. On the other hand, the Others DU vs. Others DS contrast revealed activation of the right extrastriate body area (EBA) and left superior parietal lobe (SPL) (Table 2. Fig. 3B).

**Table 2:** Brain activity in the DU vs. DS contrast for self and others.

Self DU vs. Self DS	
L/R	Brain region	BA	MNI coordinates			voxels	T-value
			x	y	z		
R	PMd	4	22	−20	80	14	4.88
R	PIVC/TPJ	40/41	66	−42	18	223	5.86
		40	54	−40	34		5.4
R	IPL	40	54	−62	46	11	5.69
L	aSMG	40	−58	−34	36	10	4.14
R	fusiform gyrus	19	48	−76	−12	49	4.29
		19/37	44	−68	−14		4.22
L	fusiform gyrus	37	−38	−70	−18	10	5.48
R	putamen		30	10	2	10	4.46
R	caudate nucleus		24	6	22	23	7.37

Others DU vs. Others DS	
L/R	Brain region	BA	MNI coordinates			voxels	T-value
			x	y	z		
L	SPL	7	−16	−68	60	18	4.13
R	EBA	37	56	−60	−12	449	7.04
		37	52	−70	−4		6.45

Brain regions significantly activated for the Self DU vs. Self DS contrast (upper) and Others DU vs. Others DS contrast (lower).

BA: Brodmann area, MNI: Montreal Neurological Institute, PMd: dorsal premotor area, PIVC: parieto-insular vestibular cortex, TPJ: temporo-parietal junction, IPL: inferior parietal lobe, aSMG: anterior supramarginal gyrus, SPL: superior parietal lobe, EBA: extrastriate body area.

### Self-specific neural activity in the DU vs. DS contrast

**Figure 4:**
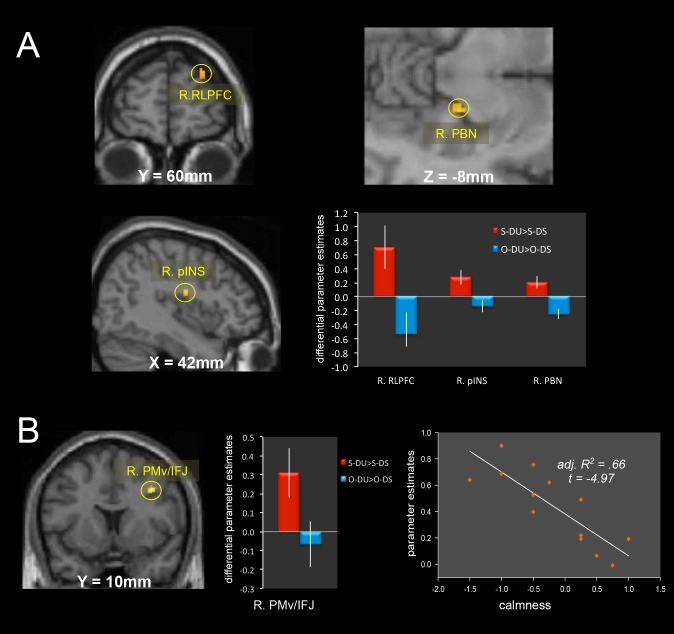
Self-specific brain activity in the DU vs. DS contrast. **A:** Brain regions significantly activated for the Self (DU > DS) vs. Others (DU > DS) contrast, and the differential (Self DU (S-DU) vs. Self DS (S-DS), Others DU (O-DU) vs. Others DS (O-DS)) eigenvariate values (parameter estimates, mean ± standard error) in the spherical ROI (radius, 5 mm) whose center was the peak voxel at each cluster showing significant activity in the above contrast. **B:** IFJ/PMv significantly activated for the Self (DU > DS) vs. Others (DU > DS) contrast and its differential eigenvariate values in the spherical ROI (radius, 5 mm) whose center was the peak voxel at each cluster showing significant activity in the above contrast. In addition, this activity showed a negative correlation with subjective calmness ratings. Adj. R^2^: adjusted R^2^, RLPFC: rostral lateral prefrontal cortex, PMv: ventral premotor area, IFJ: inferior frontal junction, PBN: parabrachial nucleus.

As shown in Table 3 and Fig. 4, the contrast of (Self (DU vs. DS) vs. Others (DU vs. DS)) revealed activation of the right rostral lateral prefrontal cortex (RLPFC), inferior frontal junction/ventral premotor cortex (IFJ/PMv), posterior insular cortex and parabrachial nucleus (PBN), and the left lingual, fusiform and parahippocampal regions. Moreover, all of the average ROI eigenvariates in the contrast of Self (DU vs. DS) were positive. Among the above brain regions, IFJ/PMv, posterior insula, and PBN were considered to be specifically the possible regions corresponding to the genuine bodily instability based on the previous related studies (see “Self-specific activity during body instability processing” in Discussion). In addition, right IFJ/PMv activity was negatively correlated with calmness differential scores (adjusted R^2^ = 0.66, t = −4.97, *p* = 0.00040<0.001; Kolmogorov-Smirnov Z = 0.59, *p* = 0.88>0.05; the Durbin-Watson statistic is 2.47) (Fig. 4B). There were no other significant correlations between brain activity and subjective scores.

**Table 3:** Self-specific brain activity in the DU vs. DS contrast.

Self (DU vs. DS) vs. Others (DU vs. DS)					
L/R	Brain region	BA	MNI coordinates			voxels	T-value	ROI eigenvariates (mean ± S.E.)	
			x	y	z			Self (DU vs. DS)	Others (DU vs. DS)
R	RLPFC	10	28	60	32	21	4.47	0.70±0.31	−0.54±0.18
R	IFJ/PMv	9	36	10	38	19	5.71	0.31±0.13	−0.066±0.12
L	lingual gyrus	19	−22	−72	0	15	5	0.26±0.12	−0.21±0.092
L	fusiform gyrus	20	−44	−36	−20	36	6.98	0.24±0.076	−0.22±0.074
		37	−40	−48	−4	14	4.47	0.26±0.085	−0.17±0.11
R	pINS	14	42	−6	22	19	4.76	0.28±0.11	−0.15±0.080
R	PBN		18	−24	−8	15	4.9	0.21±0.090	−0.26±0.064
L	parahippocampal gyrus		−32	−50	8	37	4.88	0.21±0.074	−0.37±0.10

Brain regions which were significantly activated for the Self (DU vs. DS) vs. Others (DU vs. DS) contrast and whose averaged ROI eigenvariate for the Self (DU vs. DS) contrast was positive. The ROI eigenvariates including those for the Others (DU vs. DS) contrast are shown for illustration.

BA: Brodmann area, MNI: Montreal Neurological Institute, RLPFC: rostrolateral prefrontal cortex, PMv: ventral premotor area, IFJ: inferior frontal junction, pINS: posterior insula, PBN: parabrachial nucleus.

## Discussion

### The neural basis of processing body instability

In the self-condition, the brain regions activated during perception of a dynamically unstable state are involved in extracting and processing unstable components of whole body movement. In monkeys, the PIVC at the posterior end of the insula constitutes the core region of the vestibular cortex, as it contains many vestibular-driven neurons [26], [27], [28]. The PIVC is also considered to be the core region of the vestibular cortex in humans [27], [29], [30], [31] and receives disynaptic inputs from the vestibular complex via the thalamus [32], [33]. PIVC activity during vestibular stimulation is stronger in the right hemisphere in right-handers [34], in concordance with the present findings. In addition, right peri-sylvian areas including the IPL are also related to vestibular functioning in humans (caloric or galvanic) [27], [29], [30], [35] and the PIVC is also connected with the pulvinar area, suggesting possible routes for visual inputs pertaining to body instability to the vestibular cortex [27]. In addition, the right TPJ, which partially overlaps with the PIVC, receives somatosensory, visual, and vestibular inputs, plays a critical role for encoding spatial aspects of bodily self-consciousness [36] and is activated by any salient changes in sensory stimuli [37]. Thus, activity of this region may be related to information processing of the spatial aspects of highly salient and potentially dangerous bodily movements.

There was also significant activation of the right PMd (corresponding to the lower extremities and trunk), caudate, putamen, and left aSMG. These brain regions may be involved in automatically and rapidly transforming information regarding one's unstable movements from the visual allocentric space to the egocentric motor/body spaces, based on one's own body-schema [27], [38]. A meta-analysis of the functional neuroimaging studies of action representations [39] illustrates that extensive activity overlap exists between the motor-related brain areas during action observation, simulation, and execution. Moreover, the SMG is an important node in the network of fronto-parietal sensorimotor-related areas that represent limb movements [40]. The left SMG is particularly active during a variety of tasks involving tools [39], [41] and spatiotemporal control of skilled actions [42], and plays a key role in representing memories for skilled praxis [41], [43], suggesting that the left SMG underlies body-schema representation. In addition, the left aSMG changes rapidly for the optimization of responses to vestibular input during whole-body perturbations [44], suggesting that the body-schema is flexible and can adapt to novel environments. Based on these considerations, the neural basis of the processing of one's own body instability appears to consist mainly of the following three processes. First, there is a visual process for extracting dynamic body instability, in which body instability is extracted from visual representations of one's own whole-body movements. Secondly, there is a motor/body process for space transformation (allocentric → egocentric), in which the instability components are interpreted as one's own unstable bodily state based on one's own body schema. These two processes are associated with fusiform regions, the PMd, SMG, putamen, and caudate. Finally, there is3) a vestibular process in which degree of body instability is estimated via the PIVC.

In contrast, the right EBA and left SPL were significantly activated in the Others DU vs. Others DS contrast, and these areas appear to be involved in processing others' body instability. The right EBA, which is activated strongly and selectively in response to static and dynamic images of human bodies and body parts [45], [46], [47], is activated to a greater extent by allocentric than egocentric views [48], [49], [50], and responds more to impossible than possible movements [51]. This activity might be required for the visual analysis of others' body instability, in agreement with previous findings that the recognition of others is related to visual processing, whereas recognition of the self is more related to motor processes [19]. In addition, left SPL activity is thought to be critical in the visual analysis of others' instability via the processing of specific body parts [52].

### Self-specific neural activity during body instability processing

We expected that brain regions related to homeostatic processes might be involved in self-specific body processing, given that one's sense of self is critical for survival. As expected, activation of the right PBN and posterior insula was observed during the processing of one's own bodily instability. The communication between vestibular nuclei and the PBN is bidirectional, suggesting that the discharge of some vestibular nucleus neurons may represent contextual information regarding the level of danger indicated by incoming gravito-inertial information [53]. The PBN contains cells that respond to body rotation and position relative to gravity, and it appears to be an important node in a primary network that processes convergent vestibular, somatic, and visceral information to mediate avoidance conditioning, anxiety, conditioned fear responses, and affective responses, including panic associated with falling [53]. The response properties of PBN units are appropriate for a sensory signal to detect anomalies in head stability control, as a consequence of body postural control loss relative to gravity [54]. In the present study, self-specific PBN activity during the processing of one's own body instability might evoke such responses to dangerous departures from normal and stabilized movement trajectories. While the vestibular information for discriminating signals reflecting whole body trajectory changes may contribute to either postural control or adaptive cardiovascular (e.g., vestibule/sympathetic) responding through descending PBN connections to the vestibular nuclei, medulla, and spinal cord [55], [56], inertial guidance monitoring may provide interoceptive information to ascending pathways from the PBN ipsilaterally to the insula via the thalamus. The insular cortex is organized in a hierarchical caudal–rostral direction, whereby primary sensory inputs projecting to the posterior insula, including somatosensory, vestibular and visceral inputs, are progressively elaborated and integrated across modalities in the middle insula [50], [57]. The insula differentiates sympathetic and parasympathetic activity [58], [59], and electrical stimulation of the right insular cortex elevates diastolic blood pressure and heart rate while stimulation of the left insula decreases heart rate [60], [61]. Sympathetic activity appears to be represented in the right hemisphere [58], [61], suggesting high sympathetic activity specific to one's own body instability. While there was clear evidence of self-specific brain activity, each of the subjective ratings assessed here showed no significant differences between the self and others in each of the dynamically unstable and stable conditions (Table 1, Fig. 2) and none of the DU vs. DS contrast differential ratings showed significant differences between self and other. Individuals may not be conscious of affect associated with the processing of bodily instability, based on the fact that posterior insula activation is related to unconscious processes.

A meta-analysis shows that IFJ/PMv [62] activity is associated with interpretation of potentially threat-related stimuli [63], [64]. In particular, perceiving fear during dynamic body expression induces right PMv activity [65]. Moreover, electrical stimulation of the dorsal polysensory area of the PMv evokes a specific set of defensive movements (avoiding, protecting, and withdrawing) [66]. The centering movement of the eyes that occurs during defensive reactions is evoked by stimulation of the polysensory zone sites [67]. One major function of the polysensory neurons may be to monitor nearby potentially threatening objects and to coordinate complex movements to protect the body surface from those objects, implicating involvement of the right IFJ/PMv in motor preparations/simulation for such defensive reactions to an impending bodily crisis. In fact, activity in this region showed a significant negative correlation with subjective feelings of calmness (Fig. 4B). In addition, previous studies have suggested that a defining function of the rostrolateral prefrontal cortex (RLPFC) is meta-cognitive processing [68], or the process of reflecting upon one's own mental contents [69], [70], [71], [72]. In the present study, our participants were supine in the MRI scanner and viewed video of themselves and others making potentially unstable and dangerous movements. Metacognitive processing might be required for processing one's own movements but not those of others. Additionally, the RLPFC is involved in motor learning, such that significant gray matter volume increases and fractional anisotropy decreases were observed in the RLPFC following only two sessions of practice at a complex whole-body balancing task [44].

Based on these considerations, the self-specific neural processing of body instability consists mainly of three component processes: 1) a vestibular/interoceptive process, which is related to detection of vestibular anomalies and to sympathetic activity as a form of alarm response (the right PBN and posterior insula), 2) an automatic motor-response preparation process (right IFJ/PMv), in which the necessary motor responses are automatically prepared/simulated in the brain to protect one's own body, and 3) a meta-cognitive process (right RLPFC) for self-recognition from the 3rd person perspective view. Among these components and corresponding brain regions, the right PBN, posterior insula, and IFJ/PMv are thought to be activated during the genuine experience of an unstable bodily state, together with the right PIVC, which is involved in degree of body instability estimates. In addition, all of the neural structures showed remarkable right dominance at both the cortical (PIVC, IFJ/PMv, and posterior insula) and brainstem (PBN) levels, the latter being directly connected to the vestibular nerve and therefore comprising a very primitive neural structure. This right dominance may be based on lateralization of homeostatic brain structures and functions, which has been evolutionarily driven by a preexisting behavioral and autonomic asymmetry that is present in all vertebrates [73].
